# Oral manifestation of Goltz-Gorlin syndrome in a young girl

**DOI:** 10.1186/1746-160X-8-S1-P8

**Published:** 2012-05-25

**Authors:** M Callea, I Yavuz, L Deroma, M Montanari, G Clarich, M Maglione, E Albertini, L Garavelli

**Affiliations:** 1 Department of Maxillo-Facial Surgery and Paediatric Dentistry, Institute for Maternal and Child Health, Trieste, Italy; 2 Dicle University, Diyarbakır, Turkey; 3 University Hospital Santa Maria della Misericordia, Udine, Italy; 4 University of Bologna, Italy; 5 University of Trieste, Italy; 6 University of Ferrara, Italy; 7 S. Maria Nuova Hospital, Reggio Emilia, Italy

## Introduction

Focal dermal hypoplasia (Goltz-Gorlin syndrome) is a multi-system disorder characterized by involvement of skin, skeletal system, eyes and face. It is caused by loss-of-function mutations in the *PORCN* gene. We report the case of a young female, focusing on the dental features.

## Aim

To describe the oral manifestation of a rare disorder that resembles ectodermal dysplasia (ED).

## Case report

Clinical, radiological and genetic findings revealed common features of Goltz-Gorlin syndrome and pure ED. Oro-dental characteristics of the patient mostly corresponded to those described in the literature. However, previously unreported oro-dental findings such as taurodontism, peg-shaped teeth and microdontia are considered unusual for Goltz-Gorlin syndrome, but similar to the dental features of hypohidrotic ED. Clinical characterization of the patient by a multidisciplinary approach is described and a comprehensive review of the literature is presented.

